# Broxyquinoline enhances antibacterial activity of colistin and attenuates LPS-induced inflammation

**DOI:** 10.1128/aac.01398-25

**Published:** 2026-02-03

**Authors:** Rui Ding, Kelong Ma, Kaiyao Zhang, Jiayang Liu, Yonglin Zhou, Lei Xu, Hongtao Liu, Xuming Deng, Jiazhang Qiu, Shizhen Ma

**Affiliations:** 1 State Key Laboratory for Diagnosis and Treatment of Severe Zoonotic Infectious Diseases & Key Laboratory of Zoonosis Research, Ministry of Education, Institute of Zoonosis, College of Veterinary Medicine, Jilin University 12510, Changchun, Jilin, People's Republic of China; University of Houston, Houston, Texas, USA

**Keywords:** colistin, antimicrobial adjuvant, broxyquinoline, anti-inflammatory

## Abstract

Colistin is considered one of the last-resort antibiotics for treating infections caused by multidrug-resistant (MDR) Gram-negative bacteria. However, the emergence and dissemination of mobile colistin resistance gene, *mcr*, have severely compromised the clinical utility of colistin. Combination therapy has emerged as a promising strategy to restore and enhance antibiotic efficacy against such bacterial infections. In this study, we identified broxyquinoline (BRO), an antiprotozoal compound, as a potent colistin adjuvant that significantly enhanced colistin activity against both colistin-susceptible and colistin-resistant Gram-negative bacteria by markedly reducing the minimum inhibitory concentration. Mechanistically, BRO disrupts bacterial membrane integrity, increases membrane permeability and fluidity, collapses the proton motive force, induces reactive oxygen species (ROS) accumulation, and depletes intracellular ATP, collectively disturbing bacterial homeostasis. Additionally, BRO exhibited high-affinity binding to lipopolysaccharide (LPS) and attenuated subsequent LPS-induced inflammatory responses in host cells. In murine thigh and lung infection models, the BRO-colistin combination restored colistin efficacy *in vivo*, evidenced by significantly reduced bacterial loads. In the lung infection model, this combination further improved survival, alleviated pulmonary pathological damage, and reduced the levels of pro-inflammatory cytokines (TNF-α, IL-1β) in bronchoalveolar lavage fluid. Collectively, these findings support the BRO-colistin combination as a promising therapeutic strategy to overcome colistin resistance and combat MDR Gram-negative infections.

## INTRODUCTION

Antibiotics have long played a critical role in combating bacterial infections (1). However, the global rise of multidrug-resistant (MDR) and extensively drug-resistant (XDR) bacteria has severely compromised their clinical efficacy (2–5). In this context, colistin, a non-ribosomal peptide antibiotic, has re-emerged as one of the last-resort antibiotics for treating infections caused by MDR and XDR Gram-negative bacteria, particularly carbapenem-resistant Enterobacteriaceae. Colistin exerts its bactericidal activity by binding to lipopolysaccharide (LPS) in the outer membrane, leading to disruption of membrane permeability and subsequent leakage of intracellular contents (6). However, the emergence of plasmid-mediated mobile colistin resistance gene, *mcr-1*, in 2015 and its variants has posed a severe threat to the clinical utility of colistin. The *mcr* genes encode a phosphoethanolamine transferase that modifies lipid A in LPS, thereby reducing colistin binding affinity and conferring resistance (5). The widespread dissemination of *mcr* genes poses a substantial risk to the clinical use of colistin, and effective strategies are urgently needed to overcome colistin resistance.

Given the high cost and prolonged timelines associated with the development of new antibiotics, drug repurposing and combination therapies have emerged as attractive alternative strategies (7, 8). A range of non-antibiotic agents, such as β-lactamase inhibitors (e.g., clavulanate and avibactam) (9), statins (10), and loperamide (11), have exhibited adjuvant effects in combination with various conventional antibiotics by targeting specific bacterial defense mechanisms. In particular, several colistin adjuvants have been identified: PBT2, originally developed for neurodegenerative diseases (Alzheimer’s), restores colistin susceptibility by disrupting bacterial metal ion homeostasis (12); silver nanoparticles have been found to inhibit MCR-1 by irreversibly displacing zinc ions from its catalytic site (13); melatonin enhances colistin activity by compromising the membrane permeability of Gram-negative bacteria (14); and the anthelmintic drug niclosamide restores colistin susceptibility in resistant *Klebsiella pneumoniae* by disrupting bacterial membrane integrity (15). These findings highlight the viability of combination strategies for prolonging the therapeutic lifespan of colistin.

Despite these advances, current antimicrobial strategies and agents remain inadequate to meet the prevention and control demands posed by MDR and XDR bacteria. Moreover, colistin-induced bacterial lysis can also lead to the release of free LPS from the outer membrane, thereby triggering excessive host inflammatory responses, potentially leading to sepsis and septic shock (16–18). Notably, there is a paucity of research on adjunctive therapies that both potentiate colistin activity and mitigate the pathological consequences of LPS release. Broxyquinoline (BRO), a derivative of 8-hydroxyquinoline approved by the US Food and Drug Administration (FDA), exhibits antifungal (19), antiprotozoal (20, 21), and antibacterial activities (22), with clinical trials supporting its safety and therapeutic potential (23–25). Our preliminary findings further indicated that BRO not only acted as an effective adjuvant to colistin against MDR Gram-negative bacteria but also exhibited strong LPS-binding activity that attenuated LPS-induced inflammation. In this study, we systematically investigated the adjuvant antibacterial activity of the BRO-colistin combination and elucidated its underlying mechanisms. Our findings may provide potential therapeutic strategies to overcome colistin resistance and reduce LPS-associated toxicity.

## MATERIALS AND METHODS

### Bacterial strains and reagents

The bacterial strains used in this study are listed in Table 1. Unless otherwise specified, isolates were cultured at 37°C in Luria-Bertani broth (LB; Sigma) or on LB agar plates. Murine alveolar macrophage (MH-S) cells were maintained in RPMI 1640 medium supplemented with 10% heat-inactivated fetal bovine serum (FBS; Invitrogen) and 1% (w/v) penicillin-streptomycin. All antibiotics were procured from Meilunbio (Dalian, China).

**TABLE 1:** Adjuvant effects of BRO in combination with colistin against various bacterial strains^*a*^

Species	Source	BRO MIC (μg/mL)	MIC (μg/mL)		FIC index
			Alone	Combination with BRO (32 μg/mL)	
*E. coli* ATCC 25922	Laboratory strain	1,024 ± 0.00	1.25 ± 0.43	0.22 ± 0.05	0.22 ± 0.06
*E. coli* DH5α	Laboratory strain	256 ± 0.00	0.63 ± 0.22	0.13 ± 0.00	0.34 ± 0.05
*E. coli* DH5α(pME6032-*mcr-1*)	Laboratory strain	256 ± 0.00	32 ± 0.00	0.5 ± 0.00	0.14 ± 0.00
*E. coli* B2	Laboratory strain	256 ± 0.00	0.63 ± 0.22	0.17 ± 0.02	0.17 ± 0.02
*E. coli* DZ-12R	Chicken cloacae	128 ± 0.00	20 ± 6.93	0.31 ± 0.11	0.27 ± 0.00
*E. coli* 109-11	Chicken cloacae	256 ± 0.00	1,024 ± 0.00	1.50 ± 0.50	0.13 ± 0.00
*K. pneumoniae* ZJ02	Hospital-isolated strain	2,048 ± 0.00	48 ± 16.00	1.50 ± 0.50	0.05 ± 0.00
*K. pneumoniae* E8.31	Chicken cloacae	2,048 ± 0.00	48 ± 16.00	1.50 ± 0.50	0.05 ± 0.02
*K. pneumoniae* L18	Chicken cloacae	1,024 ± 0.00	56 ± 13.86	1.25 ± 0.43	0.05 ± 0.01
*K. pneumoniae mcr*-613	Chicken cloacae	128 ± 0.00	128 ± 0.00	2.00 ± 0.00	0.27 ± 0.00
*K. pneumoniae* 111-21	Chicken cloacae	2,048 ± 0.00	1,024 ± 0.00	2.00 ± 0.00	0.02 ± 0.00
*K. pneumoniae* 13b5	Chicken cloacae	1,024 ± 0.00	20 ± 6.93	1.50 ± 0.50	0.12 ± 0.04
*S. typhimurium* HYM2	Chicken cloacae	1,024 ± 0.00	16 ± 0.00	1.00 ± 0.00	0.09 ± 0.00
*S. typhimurium* GP-9	Chicken cloacae	2,048 ± 0.00	20 ± 6.93	1.25 ± 0.43	0.09 ± 0.03
*S. typhimurium* 15E464	Hospital-isolated strain	1,024 ± 0.00	24 ± 8.00	0.75 ± 0.25	0.06 ± 0.00
*P. aeruginosa* TL-1671	Hospital-isolated strain	2,048 ± 0.00	14 ± 3.46	1.75 ± 0.43	0.16 ± 0.07
*P. aeruginosa* TL-3086	Hospital-isolated strain	2,048 ± 0.00	32 ± 0.00	2.00 ± 0.00	0.08 ± 0.00

^*a*^ All data represent the mean ± SD of three independent biological replicates.

### Determination of MICs

The MICs of the compounds were determined using the broth microdilution method as described previously (26). Briefly, compounds were twofold serially diluted in sterile 96-well microtiter plates, and an equal volume of bacterial suspension was added to achieve a final concentration of 5 × 10^5^ CFU/mL. Plates were incubated at 37°C for 18 h, and the MIC was defined as the lowest concentration showing no visible bacterial growth.

### Assessment of BRO effects on antibiotics by checkerboard assay

The interaction effects between antibiotics and BRO were assessed using a checkerboard assay. Briefly, 100 μL of LB broth was dispensed into each well of a 96-well microtiter plate. Antibiotics and BRO were twofold serially diluted along the horizontal and vertical axes, respectively. Subsequently, 100 μL of bacterial suspension (1 × 10^6^ CFU/mL) for each strain (*E. coli* DH5α(pME6032-*mcr-1*), *E. coli* B2, *S. typhimurium* HYM2, and *K. pneumoniae* ZJ02) was added to each well. Plates were incubated at 37°C for 18 h, and optical density at 600 nm (OD_600_) was measured. Synergy was defined as the fractional inhibitory concentration index (FICI) ≤ 0.5, calculated using the formula provided previously (27).

### BRO–colistin antibacterial activity evaluated by time-kill and disk diffusion assays

Overnight cultures of *E. coli* DH5α(pME6032-*mcr-1*), *E. coli* B2, *S. typhimurium* HYM2, and *K. pneumoniae* ZJ02 were diluted in LB broth to 5 × 10^5^ CFU/mL and treated with colistin, BRO, or their combination. For time-kill analysis, 100 μL aliquots were collected every 2 h over a 24-h period, serially diluted in sterile PBS, plated on LB agar, and incubated overnight at 37°C to determine colony-forming units (CFU) and bacterial survival.

Combination disk testing (CDT) was conducted as previously described (28, 29). Overnight cultures of *E. coli* B2, *S. typhimurium* HYM2, and *K. pneumoniae* ZJ02 were adjusted to OD_600_ of 0.5, diluted to an OD_600_ of 0.3 in molten LB agar, and supplemented with BRO at 0, 8, or 32 μg/mL. Ten milliliters of the mixture was poured into each plate. After solidification, a colistin disk was placed at the center of each plate, followed by pre-incubation at 4°C for 30 min and subsequent incubation at 37°C for 18–24 h. Inhibition-zone diameters were measured and compared across BRO concentrations (0, 8, and 32 μg/mL).

### Hemolysis and cytotoxicity analysis of the BRO–colistin combination

The cytotoxicity of BRO was evaluated in MH-S cells and sheep red blood cells, as previously described (30). Overnight-adherent MH-S cells were treated with varying concentrations of BRO for 12 h. Cell viability was measured using the CCK8 reagent (C0038, Beyotime) at OD_450_. For hemolysis testing, 8% (v/v) sheep red blood cells were incubated with BRO at 37°C for 1 h, with PBS and sterile water serving as negative and positive controls, respectively. After centrifugation (2,000 rpm, 3 min, 4°C), the absorbance of the supernatant was measured at OD_560_, and the hemolysis ratio was calculated.

### Assessment of bacterial membrane stability

Outer membrane integrity was evaluated using the fluorescent probe N-phenyl-1-naphthylamine (NPN; Sigma-Aldrich, Cat. No. 104043), with minor modifications to established methods (11). Overnight cultures of *E. coli* B2, *S. typhimurium* HYM2, and *K. pneumoniae* ZJ02 were diluted 1:100 in fresh LB medium and grown to OD_600_ of 0.5. Bacteria were harvested, washed three times with PBS, and resuspended in the same volume of PBS. BRO was added at various concentrations, and suspensions were incubated at 37°C with shaking for 2 h. NPN was then added to a final concentration of 10 μM, followed by a 30-min incubation. Fluorescence was measured in white opaque, flat-bottom 96-well luminescence plates (WHB-96-01) using a microplate reader (excitation: 350 nm; emission: 420 nm).

Inner membrane permeability was assessed using the fluorescent probe propidium iodide (PI; Thermo Fisher Scientific, Cat. No. P1304MP). Cultures of *E. coli* B2, *S. typhimurium* HYM2, and *K. pneumoniae* ZJ02 at an OD_600_ of 0.5 were washed and resuspended in PBS, and then treated with varying concentrations of BRO with shaking for 2 h. PI was added to a final concentration of 100 nM, and the suspension was incubated for 30 min. Fluorescence was measured at 535 nm excitation and 615 nm emission.

### Assessment of bacterial membrane fluidity

Membrane fluidity was assessed using the fluorescent probe trimethylammonium-1,6-diphenyl-1,3,5-hexatriene (TMA-DPH; MedChemExpress, CAS No. 115534-33-3), following established protocols (31). Overnight cultures of *E. coli* B2, *S. typhimurium* HYM2, and *K. pneumoniae* ZJ02 were diluted to the logarithmic phase, washed, and resuspended in PBS to an OD_600_ of 0.5. Bacteria were treated with various concentrations of BRO for 2 h, followed by the addition of TMA-DPH to a final concentration of 3 μM. After incubation at 37°C in the dark for 1 h, fluorescence was measured using a microplate reader (excitation: 355 nm; emission: 430 nm).

### Western blot analysis of MCR-1 and IκBα signaling

Western blotting was employed to assess MCR-1 expression in bacteria and phosphorylated/non-phosphorylated IκBα (p-IκBα/IκBα) in MH-S cells. Overnight cultures of *E. coli* DH5α(pME6032-*mcr-1*), *E. coli* B2, *S. typhimurium* HYM2, and *K. pneumoniae* ZJ02 were diluted to 1 × 10^8^ CFU/mL, treated with various concentrations of BRO, and incubated at 37°C with shaking for 6 h. Cultures were normalized to the same OD_600_, and bacterial proteins were extracted for SDS-PAGE and Western blot analysis (32). MH-S cells (1 × 10^6^) were seeded in six-well plates and permitted to adhere overnight. Standard LPS (1 μg/mL, Sigma, CAS No. 93572-42-0) or equivalent *E. coli* B2-extracted LPS was pre-incubated with BRO (32 μg/Ml) at 37°C for 1 h and then applied to MH-S cells for 4 h. Cells were lysed, and total proteins were extracted. Western blotting was performed using antibodies against IκBα and p-IκBα, followed by a goat anti-rabbit secondary antibody. Protein bands were visualized and quantified using ImageJ software.

### Measurement of bacterial intracellular pH

Overnight cultured bacteria were subcultured to mid-logarithmic growth phase, harvested, washed, and resuspended with PBS. *E. coli* B2, *S. typhimurium* HYM2, and *K. pneumoniae* ZJ02 were incubated with 2 μM BCECF-AM at 37°C for 30 min in the dark, and then washed three times to remove unbound probe. The labeled cells were treated with varying concentrations of BRO at 37°C for 2 h. Fluorescence was quantified at 488 nm excitation and 535 nm emission (33).

### Assessment of bacterial reactive oxygen species (ROS) accumulation

ROS accumulation in bacteria was measured using 2′,7′-dichlorodihydrofluorescein diacetate (DCFH-DA; S0033, Beyotime) following the manufacturer’s instructions. Log-phase *E. coli* B2, *S. typhimurium* HYM2, and *K. pneumoniae* ZJ02 were washed and resuspended in PBS, and then incubated with 10 μM DCFH-DA at 37°C for 30 min in the dark. After three PBS washes, the bacteria were treated with varying concentrations of BRO for 2 h. Fluorescence was quantified in 200 μL using a microplate reader (excitation: 488 nm; emission: 525 nm) (34).

### Quantification of bacterial ATP levels

Bacterial ATP levels were quantified using an enhanced ATP assay kit (S0027, Beyotime), following the manufacturer’s instructions. Overnight cultures of *E. coli* B2, *S. typhimurium* HYM2, and *K. pneumoniae* ZJ02 were diluted to log-phase, washed with PBS, and adjusted to an OD_600_ of 1 (approximately 10^9^ CFU/mL). Bacteria were then treated with various concentrations of BRO for 2 h, collected by centrifugation at 12,000 rpm at 4°C and lysed on ice. The lysates were centrifuged again, and the supernatants were mixed with ATP detection reagent. Luminescence was measured using a microplate reader (34).

### Investigation of BRO–LPS binding

The binding interaction between BRO and LPS was investigated using an Affinity ITC instrument (TA Instruments) at 25°C. Both compounds were dissolved in water at a concentration ratio exceeding 50:1. LPS was titrated into the BRO-containing cell over 20 successive injections. Binding parameters, including the equilibrium dissociation constant (Kd), stoichiometry (n), enthalpy change (ΔH), and entropy change (ΔS), were calculated from the resulting thermograms (35).

### Immunofluorescence analysis of P65 nuclear translocation

MH-S cells (1 × 10^5^) were seeded on coverslips in 24-well plates and incubated for 18 h. BRO (32 μg/mL) was pre-incubated with standard LPS or *E. coli* B2-derived LPS at 37°C for 1 h, then co-incubated with MH-S cells for another hour. Slides were washed with PBS, fixed with 4% paraformaldehyde, permeabilized with 0.2% Triton X-100, and blocked with 4% goat serum. Cells were stained with an anti-P65 primary and a fluorescent secondary antibody. Nuclei were counterstained with Hoechst for 5 min. After drying and mounting, samples were imaged using confocal laser scanning microscopy to assess P65 nuclear translocation (36).

### Detection of inflammatory cytokines

The impact of BRO on the expression of inflammatory cytokines TNF-α and IL-1β in MH-S cells and mouse bronchoalveolar lavage fluid (BALF) was assessed using enzyme-linked immunosorbent assay (ELISA). MH-S cells (10^6^ cells/well) were seeded in six-well plates and incubated overnight to allow adherence, and then treated as described in the Western blot section. After treatment, cells were harvested, centrifuged at 4°C, and the supernatants were collected. Levels of TNF-α and IL-1β were measured using ELISA kits (ABclonal Biotechnology Co., Ltd., China).

### Transcriptome analysis of BRO-treated *E. coli* B2

Overnight cultures of *E. coli* B2 were diluted 1:100 into fresh LB broth and grown to the log phase (OD_600_ = 0.5). Bacteria were harvested, resuspended in PBS, and treated with 32 μg/mL BRO or an equal volume of DMSO as a control for 4 h. Total RNA was then extracted according to the manufacturer’s instructions. cDNA library construction, sequencing, and transcriptomic data acquisition were performed by Novogene (Beijing, China) (14).

### RT-PCR analysis of gene expression

LPS was preincubated with 32 μg/mL BRO for 1 h, and then applied to MH-S cells for 4 h. Concurrently, log-phase *E. coli* B2 was treated with 32 μg/mL BRO or DMSO control for 4 h. Cells were collected by centrifugation, and total RNA was extracted using Trizol reagent (Cat#15596018; Thermo Fisher). A total of 2 μg RNA was reverse transcribed using the RevertAid RT Reverse Transcription Kit (Cat#K1691; Thermo Fisher). Quantitative RT-PCR was performed with SYBR Green dye (Cat#KTSM1401; AlpaLife, China) on an Applied Biosystems QuantStudio 1 Real-Time PCR System (Thermo Fisher). Primer sequences were listed in Table S1. Relative gene expression was calculated using the 2^−ΔΔCt^ method (37).

### Mouse infection models and therapy assessment

The *in vivo* efficacy of the BRO-colistin combination was assessed using mouse infection models according to previously published protocols (38–40).

A thigh muscle infection model was established by injecting 2 × 10^7^ CFU log-phase *E. coli* B2 into the left thigh of 40 BALB/c mice (6–8 weeks, 18–20 g) (38). One hour post-infection, mice received subcutaneous injections of PBS, colistin (8 mg/kg) (39), BRO (50 mg/kg; based on preliminary experiments, data not shown), or the BRO-colistin combination every 12 h for a total duration of 36 h. At 48 h post-infection, mice were euthanized, and the infected thigh muscles were excised, homogenized, serially diluted, and plated on LB agar for bacterial enumeration.

A pneumonia infection model was established by intranasally inoculating anesthetized female C57BL/6 mice (6–8 weeks, 18–20 g) with log-phase *K. pneumoniae* ZJ02 (2 × 10^9^ CFU for survival studies, and 2 × 10^8^ CFU for bacterial load determination, cytokine quantification, and pathological evaluations) (40). One hour post-infection, mice were treated subcutaneously with colistin (5 mg/kg) (38), BRO (50 mg/kg), or their combination. A subset of mice was treated every 12 h for 36 h and euthanized at 48 h post-infection for determination of lung bacterial loads, histopathological examination, immunohistochemical analysis, and measurement of TNF-α and IL-1β levels in BALF. Another group received three days of treatment and was monitored for seven-day survival to assess therapeutic efficacy.

## RESULTS

### BRO as a colistin adjuvant against Gram-negative bacteria

**Fig 1:**
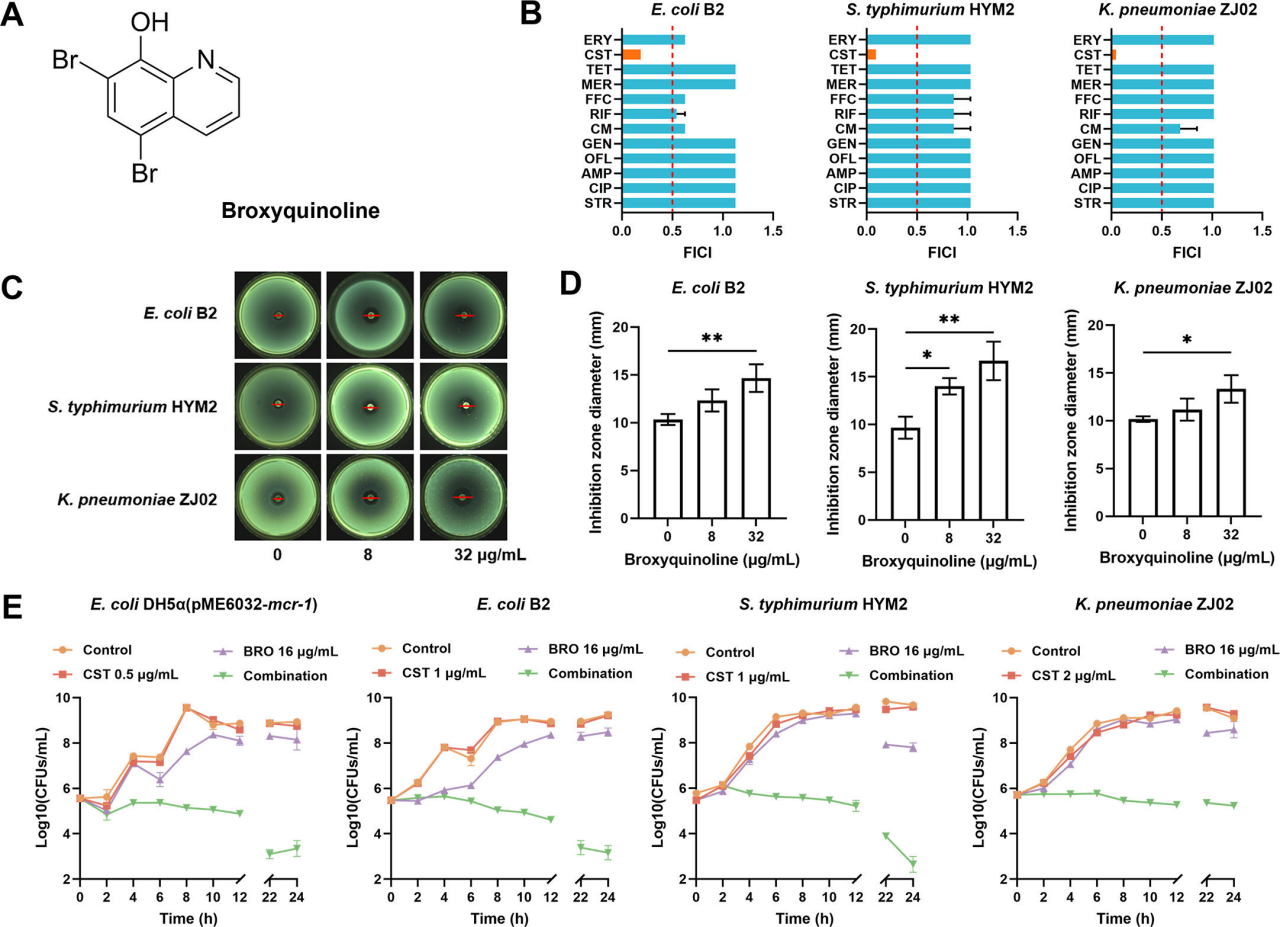
BRO enhances the bactericidal activity of colistin against Gram-negative bacteria. (**A**) Chemical structure of BRO. (**B**) The combined effects of BRO with various antibiotics against colistin-resistant *E. coli* B2, *S. typhimurium* HYM2, and *K. pneumoniae* ZJ02 were assessed using the checkerboard dilution method. Drug interactions were evaluated by calculating the fractional inhibitory concentration index (FICI), with FICI ≤ 0.5 indicating synergy. Abbreviations: ERY, erythromycin; CST, colistin; TET, tetracycline; MER, meropenem; FFC, florfenicol; RIF, rifampicin; CM, chloramphenicol; GEN, gentamicin; OFL, ofloxacin; AMP, ampicillin; CIP, ciprofloxacin; STR, streptomycin. (**C and D**) Disk diffusion assays showed inhibition zones produced by colistin (10 μg) in the presence of increasing concentrations of BRO against *E. coli B2*, *S. typhimurium* HYM2, and *K. pneumoniae* ZJ02. (**E**) Determination of the time-kill curves of *E. coli* DH5α(pME6032-*mcr-1*), *E. coli* B2, *S. typhimurium* HYM2, and *K. pneumoniae* ZJ02 treated with colistin in combination with BRO. All experiments were performed in triplicate, and data are presented as the mean ± standard deviation. Statistical significance was determined using unpaired *t*-tests or one-way ANOVA (*, *P* < 0.05; **, *P* < 0.01).

Using checkerboard dilution assays, BRO at 32 μg/mL was evaluated combined with 12 antibiotics representing diverse mechanisms of action (Fig. 1A and B). BRO exclusively enhanced the antibacterial activity of colistin against three representative colistin-resistant Gram-negative strains (*E. coli* B2, *S. typhimurium* HYM2, and *K. pneumoniae* ZJ02). BRO showed modest potentiation of florfenicol, rifampicin, and chloramphenicol against *E. coli* B2 and *S. typhimurium* HYM2 but did not reach the threshold for synergistic activity (FICI > 0.5; Fig. 1B). Concomitantly, inhibition zone assays revealed that the diameters of inhibition zones increased in a BRO concentration-dependent manner when combined with colistin (10 μg) against these wild isolates (Fig. 1C and D). In *mcr-1*-positive *E. coli* DH5α (pME6032-*mcr-1*) and the above wild isolates, BRO dose-dependently reduced the MIC of colistin and achieved complete bacterial eradication within 24 h in time-kill assays (Fig. 1E ; Fig. S1). At 32 μg/mL, BRO also enhanced the efficacy of colistin against various standard and clinical isolates (Table 1). Notably, the FICI values for *E. coli* ATCC 25922 and DH5α were 0.22 and 0.34, respectively, confirming BRO’s ability to enhance colistin efficacy in both colistin-resistant and colistin-susceptible Gram-negative bacteria.

### Hemolysis and cytotoxicity of the BRO–colistin combination

To evaluate the safety of the BRO-colistin combination, we assessed its hemolytic activity against sheep red blood cells and its cytotoxicity in MH-S cells. Neither BRO nor colistin, either alone or in combination, induced hemolysis under the tested conditions (Fig. S2A). Additionally, BRO exhibited no significant cytotoxicity in MH-S cells even at concentrations up to 128 μg/mL (Fig. S2B). The impact of diverse ionic environments on the antibacterial activity of the BRO-colistin combination was investigated to ascertain its functional stability. Varying concentrations of monovalent ions (Na^+^ and K^+^) and selected divalent ions (Zn^2+^ and Mn^2+^) did not compromise the antibacterial activity of the BRO-colistin combination. In contrast, escalating concentrations of Ca^2+^, Mg^2+^, Cu^2+^, and Fe^3+^ ions significantly attenuated or completely abolished the colistin-enhancing effect of the combination (Fig. S3). These ion-dependent effects suggest that the potentiation mechanism may be associated with membrane disruption or involve a biphasic process.

### BRO enhances colistin activity by disrupting bacterial membranes

**Fig 2:**
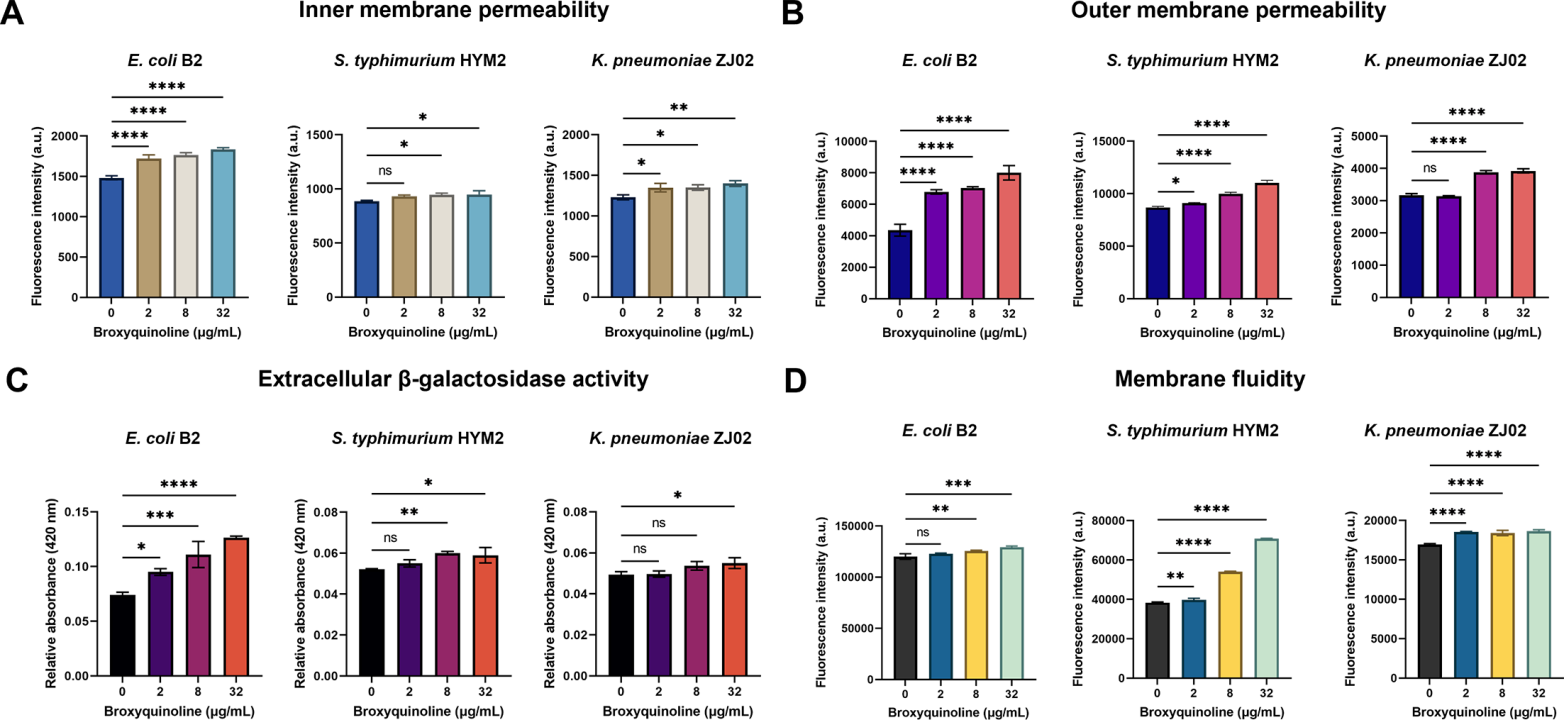
BRO disrupts bacterial membrane integrity and enhances membrane fluidity. (**A**) Inner membrane permeability was assessed by measuring propidium iodide (PI) fluorescence (excitation/emission: 535 nm/615 nm) following treatment with increasing concentrations of BRO. (**B**) Outer membrane permeability was assessed using NPN fluorescence following BRO treatment (excitation/emission: 350 nm/420 nm). (**C**) Extracellular β-galactosidase activity was measured after treatment with varying concentrations of BRO. (**D**) Membrane fluidity was assessed using the fluorescent probe TMA-DPH following BRO treatment (excitation/emission: 355 nm/430 nm). All experiments were performed in triplicate, and data are presented as the mean ± standard deviation. Statistical significance was assessed using one-way ANOVA (ns, not significant; *, *P* < 0.05; **, *P* < 0.01; ***, *P* < 0.001; ****, *P* < 0.0001).

The bactericidal effect of colistin is primarily attributed to disruption of the outer membrane through the displacement of divalent cations (41). Therefore, we hypothesized that BRO enhanced colistin susceptibility by similarly compromising bacterial membrane integrity. BRO treatment increased PI fluorescence, NPN uptake, and β-galactosidase release in a dose-dependent manner indicating disruption of both the inner and outer bacterial membranes and increased membrane permeability (Fig. 2A through C). Alterations in membrane permeability can influence membrane rigidity (42). Consistent with this observation, BRO significantly enhanced membrane fluidity in a dose-dependent manner, as measured by TMA-DPH (Fig. 2D). These structural perturbations likely weakened bacterial resistance to antibiotic intervention.

MCR-1 confers colistin resistance by modifying lipid A in LPS, thereby reducing colistin binding affinity (43). To investigate whether BRO affects MCR-1 expression, we measured MCR-1 levels in *mcr-1*-positive isolates after 8 h of treatment with BRO (≤64 μg/mL). No alteration in MCR-1 expression was observed (Fig. S4), indicating that the adjuvant effect of BRO in combination with colistin is not contingent upon changes in MCR-1 expression.

### Oxidative stress and energy metabolic disruption mediate the adjuvant effect of BRO

**Fig 3:**
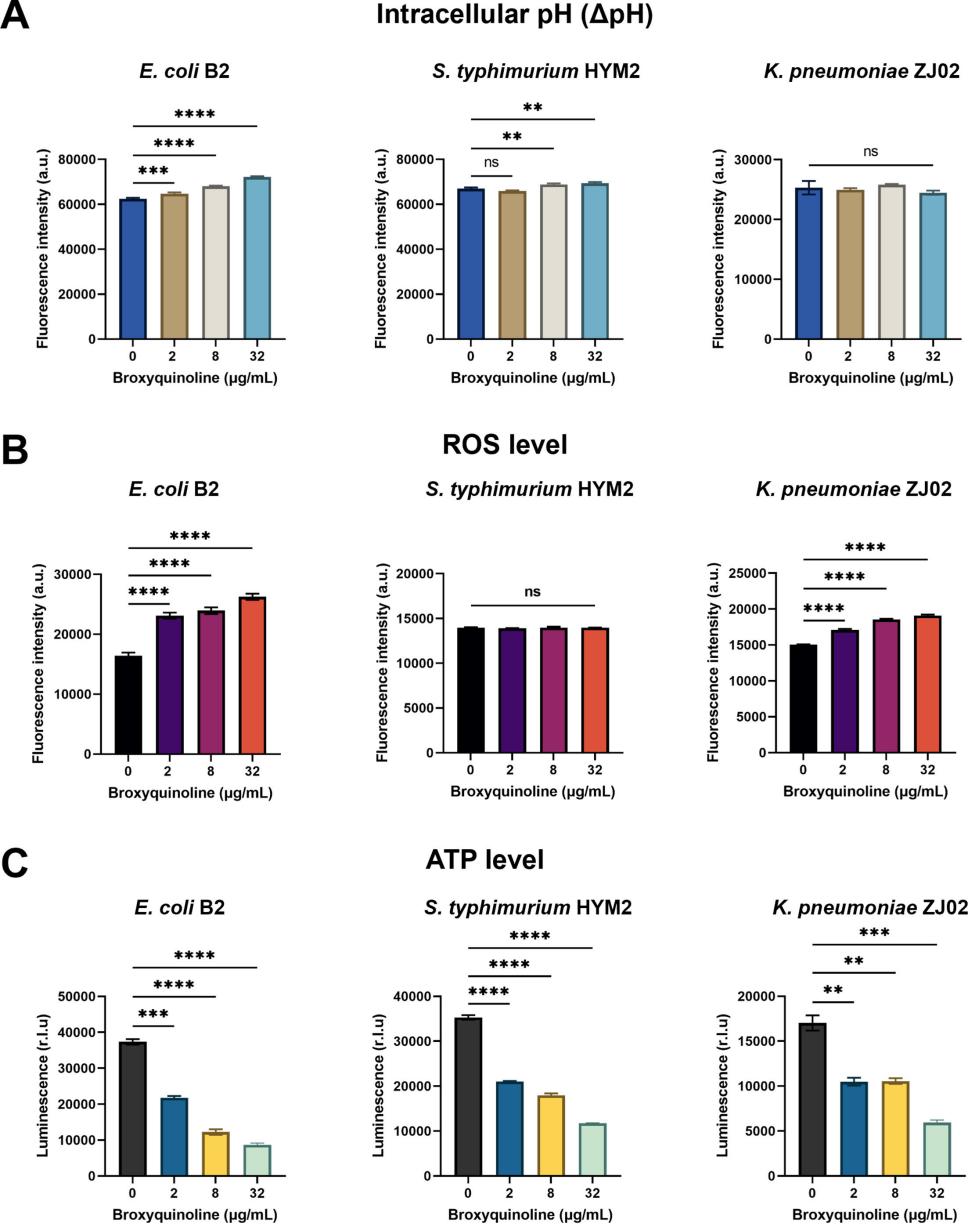
BRO treatment disrupts bacterial homeostasis. (**A**) Intracellular pH changes were assessed using the pH-sensitive fluorescent probe BCECF-AM (excitation/emission: 503 nm/530 nm). (**B**) Intracellular ROS levels were measured using a fluorescent probe (excitation/emission: 488 nm/525 nm) following BRO treatment. (**C**) Intracellular ATP levels were quantified following BRO treatment. All experiments were performed in triplicate, and data are presented as the mean ± SD. Statistical significance was determined using one-way ANOVA (ns, not significant; *, *P* < 0.05; **, *P* < 0.01; ***, *P* < 0.001; ****, *P* < 0.0001).

The bacterial membrane is essential for maintaining cell morphology and regulating material exchange, and its dysfunction disrupts cellular homeostasis. Given that BRO markedly altered membrane permeability and fluidity, we investigated its impact on intracellular homeostasis. Using the pH-sensitive probe BCECF-AM, we assessed changes in the proton motive force (PMF), a key driver of bacterial energy metabolism. BRO treatment led to an increase in the transmembrane proton gradient (ΔpH) in *E. coli* B2 and *S. typhimurium* HYM2 but not in *K. pneumoniae* ZJ02 (Fig. 3A). PMF, comprising both ΔpH and membrane potential (ΔΨ), plays a central role in maintaining intracellular equilibrium (44). However, subsequent analyses showed that BRO interacted with the ΔΨ-sensitive dye DISC3-5, resulting in fluorescence quenching and precluding accurate measurement of ΔΨ.

ROS, commonly induced by membrane damage and intracellular stress, contributes significantly to antibiotic-mediated bacterial killing (45). BRO induced a dose-dependent increase in ROS levels in *E. coli* B2 and *K. pneumoniae* ZJ02, but not in *S. typhimurium* HYM2 (Fig. 3B). Relative to the BRO-colistin combination alone, the co-administration of the antioxidant N-acetylcysteine (NAC) significantly suppressed ROS generation. This diminished ROS level was associated with a notable weakening of the BRO-colistin combination’s antibacterial activity. Consequently, a substantial increase in the number of surviving bacteria was observed following NAC addition (Fig. S5A). Furthermore, BRO-treated bacteria displayed increased sensitivity to hydrogen peroxide (Fig. S5B), underscoring impaired oxidative defenses. Consistent with these findings, BRO also caused a dose-dependent decrease in intracellular ATP levels (Fig. 3C), further disrupting cellular homeostasis.

**Fig 4:**
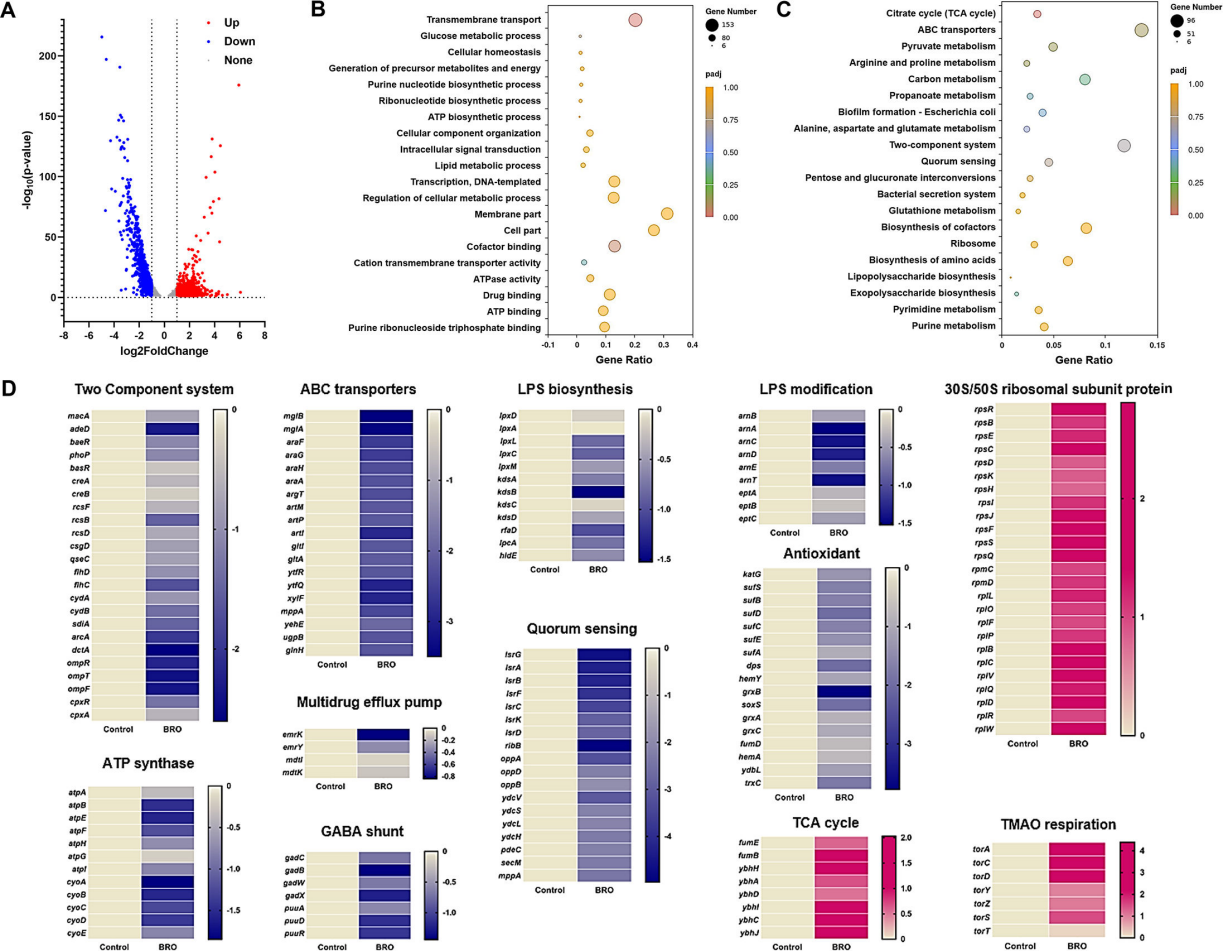
Transcriptomic profiling of BRO-treated *E. coli* B2. (**A**) A volcano plot displays differentially expressed genes (DEGs) following BRO treatment. (**B**) GO enrichment analysis was performed to classify DEGs into biological process categories. (**C**) KEGG pathway enrichment analysis was used to identify significantly affected metabolic and regulatory pathways. (**D**) Functional annotation highlights representative DEGs involved in two-component systems, ABC transporters, LPS biosynthesis and modification, ATP synthesis, multidrug efflux, the TCA cycle, and 30S/50S ribosomal subunit biogenesis.

To explore the molecular mechanisms underlying the adjuvant antibacterial activity of BRO and colistin, we performed transcriptomic analysis of *E. coli* B2 during log-phase growth. Treatment with 32 μg/mL BRO for 4 h led to the upregulation of 939 genes and the downregulation of 794 genes (|log_2_ FC| ≥ 1) compared with the DMSO control (Fig. 4A). Gene Ontology (GO) and Kyoto Encyclopedia of Genes and Genomes (KEGG) analyses revealed significant enrichment in pathways related to ABC transporters, redox regulation, two-component systems, and central metabolism (Fig. 4B and C). Upregulated genes were primarily associated with ribosomal function, the tricarboxylic acid (TCA) cycle, and trimethylamine N-oxide (TMAO) respiration, all linked to enhanced ROS production. Downregulated genes included those involved in two-component systems, ABC transporters, ATP synthesis, multidrug efflux pumps, GABA shunt, LPS biosynthesis and modification, quorum sensing systems, and antioxidative mechanisms (Fig. 4D). Suppression of ATP synthesis and LPS-related pathways may compromise energy generation, impair membrane integrity, and facilitate colistin binding. Reduced antioxidant capacity likely contributes to ROS accumulation. RT-PCR validation of selected genes confirmed the transcriptomic trends (Fig. S6).

### BRO binds to LPS and attenuates LPS-induced inflammatory responses

**Fig 5:**
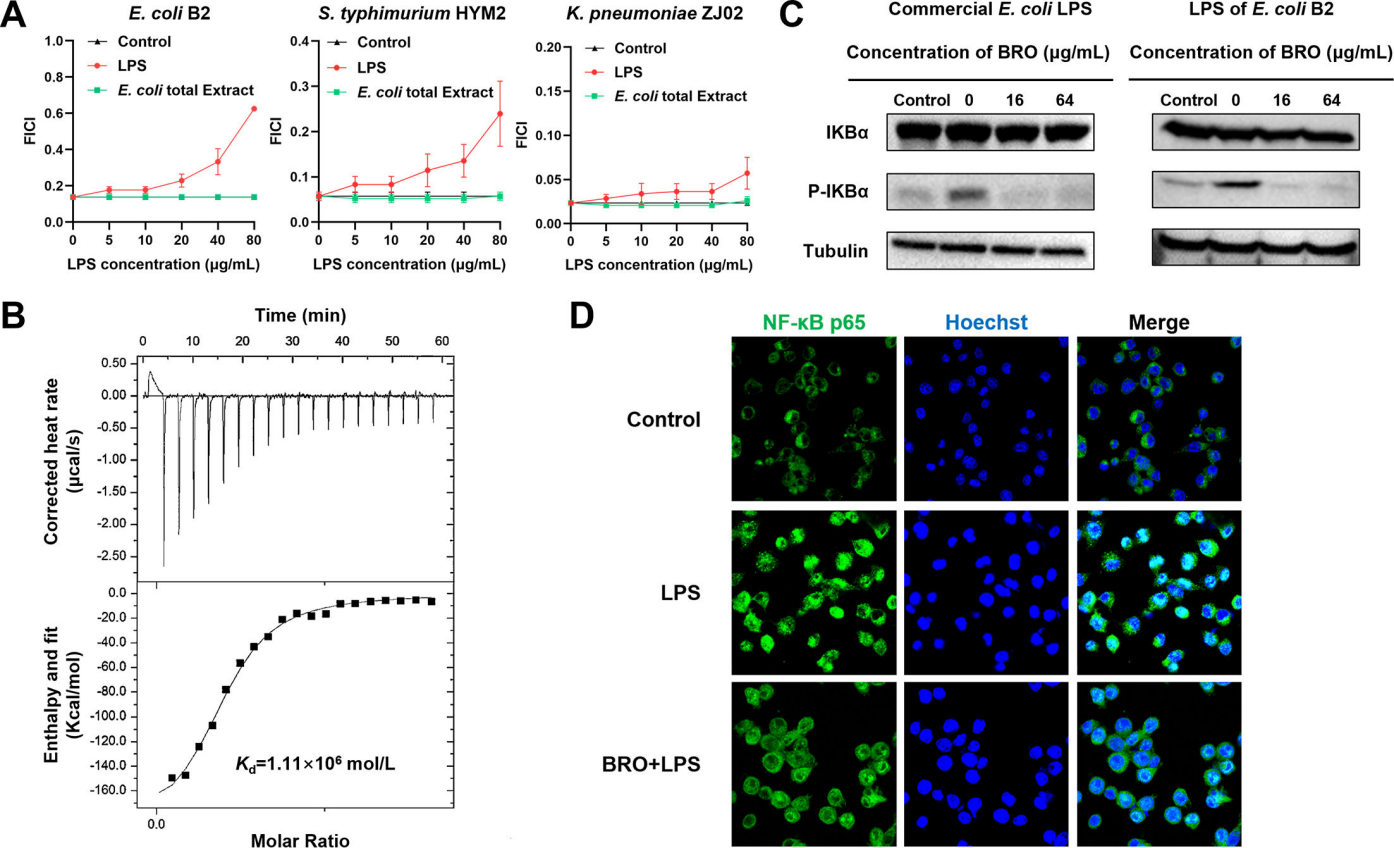
BRO neutralizes LPS and attenuates cellular inflammatory responses. (**A**) The checkerboard dilution method was used to assess the effects of exogenously added LPS and total lipid extracts from *E. coli* membranes on the synergistic bactericidal activity of BRO and colistin. (**B**) ITC analysis revealed an interaction between BRO and LPS, with thermodynamic parameters including the equilibrium dissociation constant (K_d_ = 1.11 × 10⁶ mol/L), number of binding sites (*n* = 0.100), the molar binding enthalpy (ΔH = −1.911 × 10⁵ kJ/mol), and the molar binding entropy (ΔS = −525 J/mol/K). (**C**) Western blot analysis showed that BRO supplementation markedly reduced the activation of inflammatory signaling pathways in MH-S cells stimulated with commercial *E. coli* LPS or LPS isolated from *E. coli B2*. (**D**) Confocal microscopy images show BRO-mediated inhibition of LPS-induced p65 nuclear translocation.

To identify potential bacterial membrane targets of BRO, we supplemented the BRO-colistin combination with key Gram-negative bacterial membrane components and assessed their impact on the combined antibacterial activity. Total lipid extracts from *E. coli* had no effect, whereas incremental addition of LPS markedly inhibited the adjuvant bactericidal efficacy of the BRO–colistin combination against all tested strains (Fig. 5A). Isothermal titration calorimetry (ITC) confirmed a strong binding affinity between BRO and LPS, with an equilibrium dissociation constant of K_d_=1.11 × 10^6^ mol/L (Fig. 5B). These results indicate that BRO binds to LPS, disrupts the outer membrane, and enhances the bactericidal efficacy of colistin.

Lysis of Gram-negative bacteria by antibiotics releases LPS (46), a thermostable endotoxin that persists after bacterial death and triggers host inflammatory responses (47, 48). We examined the effect of BRO on LPS-induced inflammatory by measuring IκBα phosphorylation within the canonical NF-κB pathway. Both commercial *E. coli* LPS and LPS extracted from *mcr-1*-positive *E. coli* B2 significantly induced NF-κB activation in MH-S cells within 4 h, whereas pretreatment with 16 μg/mL BRO significantly reduced this response (Fig. 5C). Moreover, BRO markedly reduced the transcription and expression of the proinflammatory cytokines TNF-α and IL-1β in MH-S cells (Fig. S7). LPS-induced inflammation is characterized by nuclear translocation of the NF-κB subunit P65. However, immunofluorescence analysis showed that BRO pretreatment markedly inhibited this translocation, with P65 predominantly retained in the cytoplasm and lacking distinct nuclear puncta (Fig. 5D). Collectively, these findings suggest that BRO effectively neutralizes extracellular LPS and suppresses LPS-induced cellular inflammatory responses.

### BRO restores colistin activity and reduces inflammation in murine infection models

**Fig 6:**
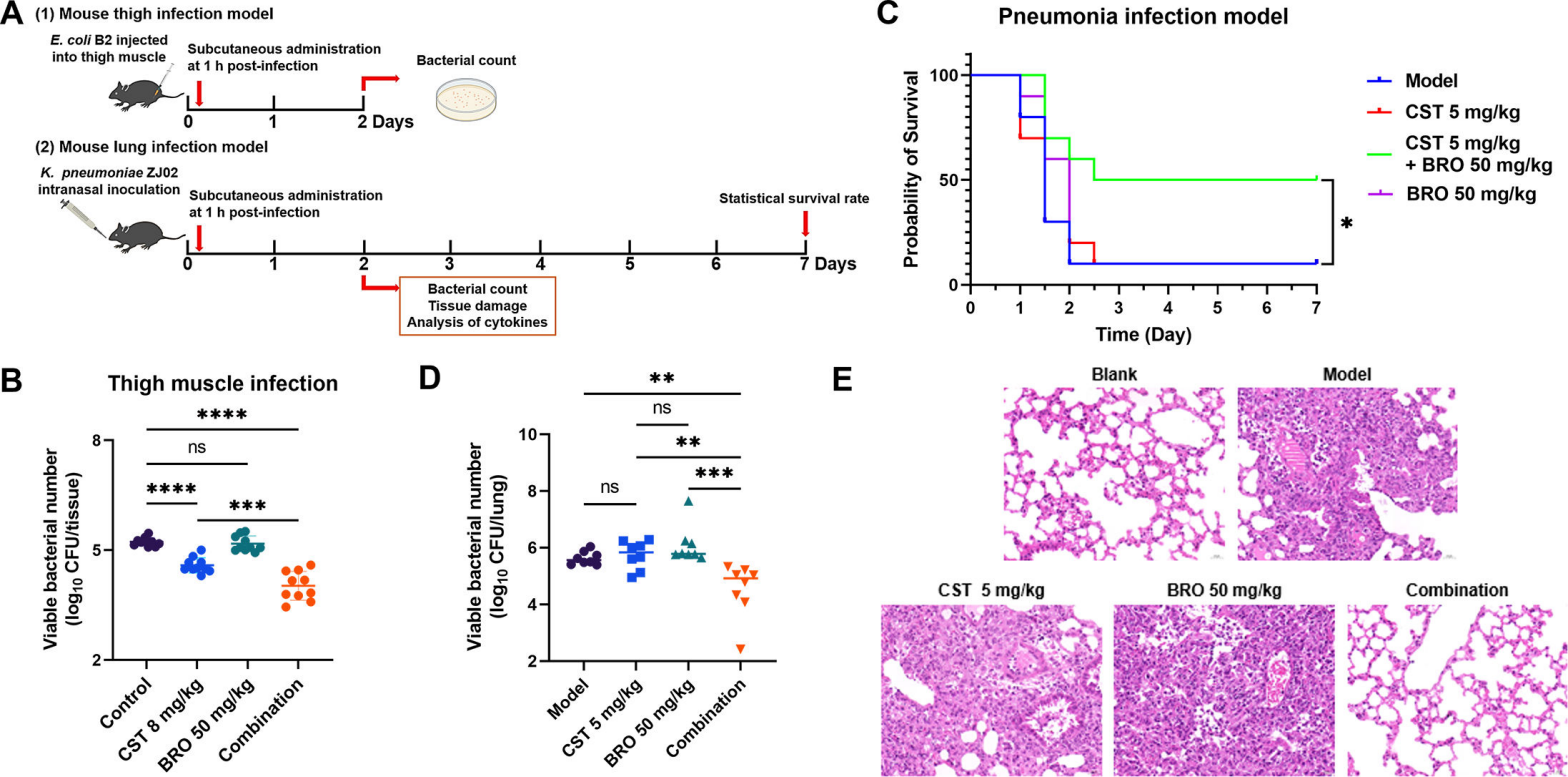
The combination of BRO and colistin exhibits potent antibacterial activity in murine infection models. (**A**) Schematic overview of the experimental design for the murine thigh muscle and pneumonia infection models. (**B**) Bacterial burdens in the thigh muscle infection model (*n* = 10). Mice infected with *E. coli* B2 and treated with colistin (8 mg/kg) or BRO (50 mg/kg) in combination with colistin (8 mg/kg) showed significantly reduced bacterial loads in muscle tissue. (**C**) Survival rates in the pneumonia infection model (*n* = 10). Combination therapy (BRO 50 mg/kg + colistin 5 mg/kg) against a lethal dose of *K. pneumonia* ZJ02 significantly improved the 7-day survival rate of infected mice. *P*-values were determined using the log-rank (Mantel-Cox) test. (**D**) Combination therapy significantly reduced bacterial loads in the lungs of infected mice (*n* = 8). (**E**) Histopathological examination of lung tissues from the pneumonia infection model following different treatments. *P*-values were calculated using one-way ANOVA among multiple groups (ns, not significant; *, *P* < 0.05; **, *P* < 0.01; ***, *P* < 0.001; ****, *P* < 0.0001).

To ascertain the *in vivo* efficacy of BRO, its antibacterial effect in combination with colistin was evaluated in two murine infection models (Fig. 6A). In a murine thigh infection model using *E. coli* B2, BRO-colistin treatment significantly reduced bacterial counts in the infected tissue compared with either agent alone (Fig. 6B). Similarly, in a pneumonia model induced by *K. pneumoniae* ZJ02, BRO-colistin treatment improved mouse survival from 10% to 50%, whereas neither agent alone achieved comparable protection (Fig. 6C). Consistently, bacterial loads in lung tissue were effectively cleared only by the combination therapy (Fig. 6D). Histopathological examination further revealed that the BRO-colistin combination markedly diminished pulmonary hemorrhage and congestion compared with PBS and single-agent treatment groups, thereby restoring lung architecture to near-healthy levels (Fig. 6E). Additionally, expression of the inflammatory cytokines IL-1β and TNF-α in the BALF of infected lungs was significantly reduced in the BRO-colistin treated group (Fig. S8). Collectively, these findings indicate that BRO can substantially restore the bactericidal activity of colistin *in vivo*, alleviate pathological damage, and improve disease outcomes in mice infected with colistin-resistant bacteria.

## DISCUSSION

Colistin is one of the last-resort therapies for MDR and XDR Gram-negative bacteria (41), yet its clinical utility is increasingly compromised by widespread *mcr*-mediated resistance (5). Given the slow pace of new antibiotic development, repurposing existing compounds as antimicrobial adjuvants represents a practical strategy (49). Several FDA-approved non-antibiotic agents, such as diclofenac sodium (50), bismuth citrate (51), and benzydamine (52), have shown antibiotic-potentiating activity. Here, we identified BRO, an antiprotozoal compound, as a potent colistin adjuvant against both colistin-susceptible and colistin-resistant Gram-negative pathogens while exerting minimal or negligible effects on the other tested antibiotics.

**Fig 7:**
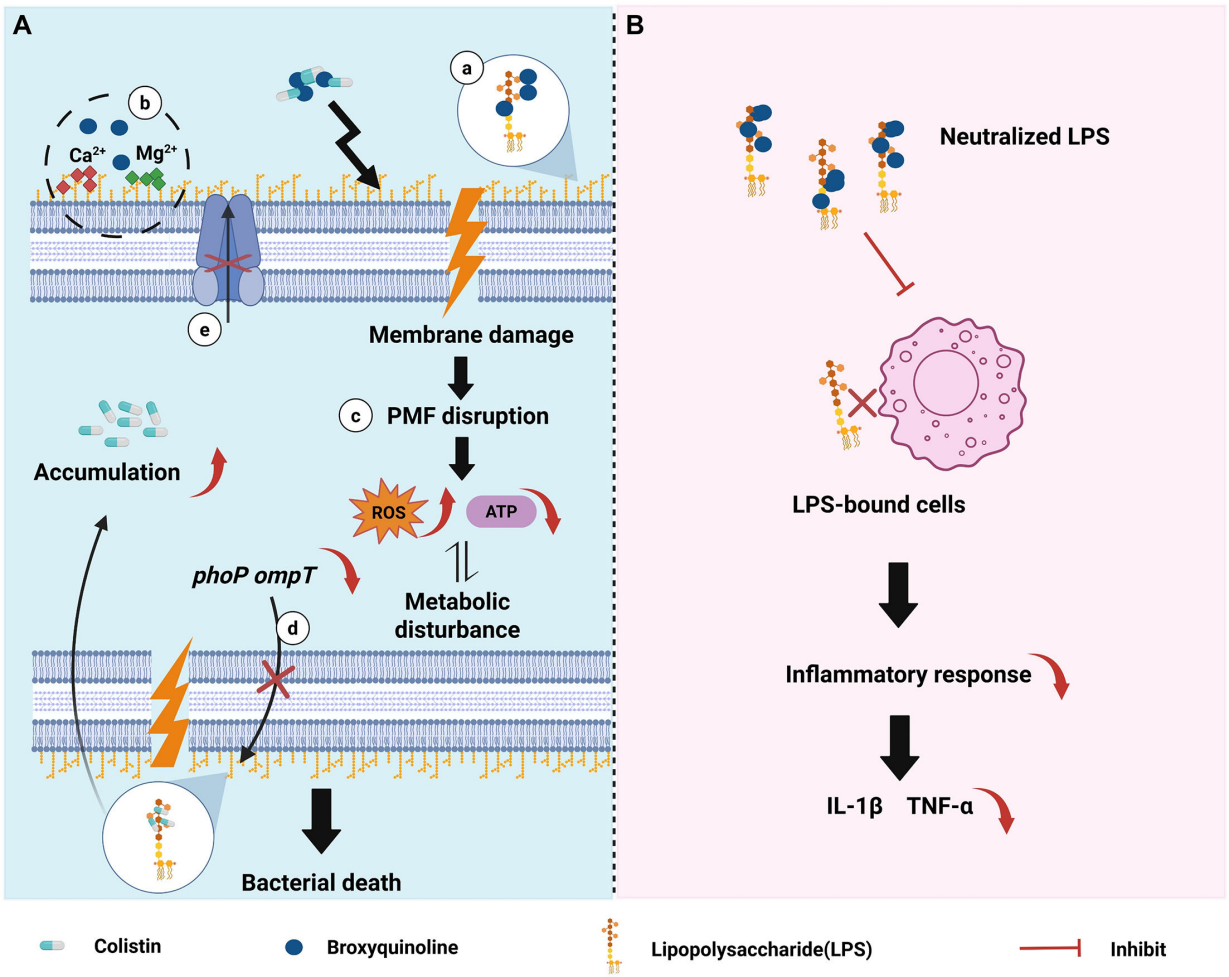
Scheme of the potential mechanisms of BRO as a colistin adjuvant. (**A**) BRO in combination with colistin exerts activity through the following mechanisms: (a) binding of BRO to LPS; (b) BRO chelates Ca²^+^ and Mg²^+^, thereby perturbing bacterial membrane; (c) membrane damage disrupts the PMF, leading to accumulation of ROS, reduced ATP production, and metabolic dysregulation; (d) transcription of *phoP* and *ompT* is downregulated, resulting in reduced LPS modification, thereby increasing the binding affinity of colistin for LPS and promoting increased intracellular accumulation of colistin; (e) efflux pump activity is inhibited. (**B**) BRO neutralizes free LPS, thereby reducing its binding to host cells and subsequently attenuating LPS-induced inflammation responses.

BRO selectively enhances colistin activity, suggesting a shared membrane-targeting mechanism. Our data demonstrate that BRO acts as a potent membrane-targeting adjuvant, disrupting both outer and inner membrane integrity (Fig. 7A), as evidenced by increased NPN and PI uptake, cytoplasmic β-galactosidase leakage, and increased membrane fluidity. Consistent with other membrane-active adjuvants, such as melatonin (14), BRO exhibits high-affinity binding to LPS, which likely disrupts hydrophobic interactions and divalent cation-mediated cross-linking between lipid A moieties. These alterations collectively disrupt membrane homeostasis and facilitate deeper penetration of colistin, providing a mechanistic basis for their combined bactericidal effect. Transcriptomic profiling further supports this membrane-centric mechanism, revealing broad downregulation of genes involved in LPS biosynthesis, lipid A modification, and outer-membrane homeostasis. As an 8-hydroxyquinoline derivative, BRO also likely retains metal-chelating capacity (53), which may contribute to its adjuvant activity. Ion-stability assays showed that exogenous Ca²^+^ and Mg²^+^ markedly reduced the antibacterial effect of the BRO-colistin combination. Given the essential role of these cations in stabilizing LPS packing (54, 55), their chelation by BRO likely intensifies lipid disorder and confers stronger membrane destabilization. This ion-dependent enhancement resembles the behavior of other cation-disrupting compounds, such as berberine (56) and artesunate (57), in the presence of chelating agents.

Cell membrane integrity is essential for bacterial viability and therefore represents a critical target for antibiotics and adjuvants. In this study, BRO markedly compromised membrane structure and fluidity, thereby disrupting ion gradients and membrane potential and ultimately causing collapse of the PMF. The loss of PMF, a characteristic consequence of membrane disruption, impaired electron transport and oxidative phosphorylation, leading to substantial depletion of intracellular ATP (58). Concurrently, BRO treatment triggered significant ROS accumulation, likely driven by respiratory chain imbalance and electron leakage following inner-membrane destabilization. Collectively, these findings indicate that BRO-induced membrane damage disrupts bacterial bioenergetics and redox homeostasis, resulting in PMF dissipation, ATP depletion, oxidative stress, and ultimately cell death. Notably, across *E. coli*, *S. typhimurium*, and *K. pneumoniae*, BRO consistently reduced ATP levels, demonstrating a conserved bioenergetic collapse despite interspecies differences. However, strain-specific variations were observed in ΔpH and ROS responses. *K. pneumoniae* exhibited milder ΔpH fluctuations, which may be attributed to its thick polysaccharide capsule that enhances outer membrane stability and surface charge shielding (59), thereby reducing the demand for ΔpH compensation following membrane perturbation. In contrast, *S. typhimurium* displayed blunted ROS accumulation, consistent with its robust oxidative-stress defense machinery, including the OxyR/SoxRS regulons, multiple catalases (*KatG*, *KatE*, *KatN*), periplasmic superoxide dismutases (SodCI/CII), and cytochrome *bd* oxidases that limit oxidative damage (60, 61). Despite these quantitative differences, all three species converged on the same fundamental outcome: BRO-induced inner-membrane disruption undermines PMF-dependent ATP synthesis, culminating in impaired energy metabolism and bacterial killing.

Beyond enhancing colistin activity, BRO also mitigated host inflammatory responses triggered by LPS (Fig. 7B). Following bacterial cell death, the rupture of outer membrane results in the release of LPS into the surrounding environment, posing a considerable risk, as LPS stimulation can drive excessive and recurrent inflammatory responses (47, 48). Cell-based assays showed that BRO reduced LPS-induced NF-κB activation and suppressed the transcription of pro-inflammatory cytokines. In murine pneumonia models, BRO–colistin combination therapy significantly improved survival and alleviated lung pathology compared with either monotherapies. This dual action, enhancing antimicrobial efficacy while attenuating host inflammation, underscores the therapeutic potential of BRO. Previous studies have reported that bacteria can amplify host inflammatory responses by actively releasing ATP or through ATP release during cell death and lysis (62). Our data further show that BRO significantly reduces intracellular ATP levels in bacterial cells, at least in part by inhibiting ATP synthesis pathways. This finding suggests that BRO may additionally alleviate host inflammatory responses by limiting bacterial ATP release.

Despite the encouraging *in vitro* findings, several limitations should be acknowledged. First, the well-documented nephrotoxicity of colistin (63) may compromise the clinical efficacy of the combination. Thus, careful dose optimization of the BRO–colistin regimen will be essential to maximize adjuvant efficacy while minimizing adverse effects. Second, the *in vivo* pharmacokinetics, tissue distribution, and metabolic stability of the BRO–colistin combination remain insufficiently characterized and require systematic evaluation. Finally, although this study outlines BRO’s multifaceted disruption of membrane homeostasis and bacterial stress-response systems, the determinants of strain-dependent potentiation and the precise potentiation mechanisms underlying the BRO–colistin interaction warrant further investigation.

In summary, BRO constitutes a novel membrane-targeting and LPS-binding anti-resistance agent that enhances colistin efficacy by destabilizing the outer membrane, dissipating the PMF, increasing ROS accumulation, reducing intracellular ATP, and attenuating LPS-induced inflammatory responses. These coordinated effects intensify bacterial physiological stress while mitigating host inflammation, supporting BRO’s potential as a promising anti-resistance adjuvant against colistin-resistant Gram-negative pathogens.

### Highlights

- BRO enhances the antibacterial activity of colistin by disrupting bacterial membrane integrity and perturbing intracellular homeostasis.
- BRO neutralizes LPS.
- BRO attenuates inflammation associated with bacterial infection.

## SUPPLEMENTAL MATERIAL

Supplemental Material aac.01398-25-s0001.docx Fig. S1 to S8; Table S1.

ASM does not own the copyrights to Supplemental Material that may be linked to, or accessed through, an article. The authors have granted ASM a non-exclusive, world-wide license to publish the Supplemental Material files. Please contact the corresponding author directly for reuse.
