# Supplementary material for: Broxyquinoline enhances antibacterial activity of colistin and attenuates LPS-induced inflammation
Source: Antimicrob Agents Chemother. 2026 Feb 3;70(3):e01398-25. doi: 10.1128/aac.01398-25 (PMC12959136; doi:10.1128/aac.01398-25)
Supplement: Supplemental Material — Fig. S1 to S8; Table S1. [file aac.01398-25-s0001.docx]

**Figure S1. Checkerboard assay of BRO combined with colistin.** A-D) Bactericidal activity of BRO in combination with colistin against colistin-resistant isolates, including *E. coli* DH5α (pME6032-*mcr-1*), *E. coli* B2, *S. typhimurium* HYM2, and *K. pneumoniae* ZJ02.

**Figure S2. Hemolysis and cytotoxicity analysis of the BRO–colistin combination.** A) Hemolytic activity in sheep red blood cells following treatment with BRO, colistin, or their combination at varying concentrations. B) Cytotoxicity in murine alveolar macrophage (MH-S) cells following exposure to BRO, colistin, or the combination over a range of concentrations.

**Figure S3. Stability of the antibacterial activity of the BRO–colistin combination in the presence of metal cations.** The checkerboard dilution assay was used to evaluate the effects of various metal cations (Na⁺, K⁺, Zn²⁺, Mn²⁺, Ca²⁺, Mg²⁺, Cu²⁺, and Fe³⁺) on the bactericidal activity of the BRO-colistin combination.

**Figure S4. BRO does not affect MCR-1 protein expression in bacteria.** Western blot analysis of MCR-1 protein levels in bacteria treated with varying concentrations of BRO at 37 °C for 6 hours.

**Figure S5. BRO-induced oxidative stress contributes to the bactericidal activity of the BRO-colistin combination.** A) The bactericidal effect of BRO-colistin combination was significantly reduced by the ROS scavenger N-acetyl-L-cysteine (NAC). B) BRO treatment increased sensitivity to H₂O₂ in *E. coli* B2 and *S. typhimurium* HYM2, but had no effect on *K. pneumonia* ZJ02. All experiments were performed in triplicate, and data are presented as the mean ± SD. Statistical significance was determined using one-way ANOVA (ns, not significant; *, *p* < 0.05).

**Figure S6. Transcriptional alterations in pathway-specific genes in *E. coli* B2 following BRO treatment.** RT-qPCR was used to validate the expression of representative differentially expressed genes (DEGs) identified by transcriptomic analysis. The housekeeping gene *danE* was used for normalization. Statistical significance was determined using an unpaired two-tailed Student’s *t*-test (*****p* < 0.0001).

**Figure S7. BRO inhibits LPS-induced inflammatory response in MH-S cells.** A) RT-qPCR analysis showed that BRO treatment significantly reduced the mRNA levels of IL-1β and TNF-α in LPS-stimulated MH-S cells. B) ELISA results demonstrated a corresponding decrease in the secreted of IL-1β and TNF-α protein in the cell culture supernatant following BRO treatment.

**Figure S8. BRO in combination with colistin exhibits significant anti-inflammatory activity in a murine lung infection model.** Levels of IL-1β and TNF-α in bronchoalveolar lavage fluid (BALF) from infected mice were quantified by ELISA. Treatment with BRO (50 mg/kg) in combination with colistin (5 mg/kg) significantly reduced the levels of pro-inflammatory cytokines (IL-1β and TNF-α) in BALF compared with control groups. *P* values were calculated using one-way analysis of variance among multiple groups (ns, not significant; **, *p* < 0.01; ***, *p* < 0.001).

**Table S1. RT-qPCR primers used in this study.**

| **Genes** | **Sequence(5`→3`)** |
| --- | --- |
| *atpB* F | TGCTGCCGTACATTGCTGAAC |
| *atpB* R | ACGCCCAGTGCCATAGACAG |
| *atpE* F | TGGGTCTGGCGGCAATCG |
| *atpE* R | CAGCAGAGGAATCAGATCAGGTTG |
| *atpF* F | CCGCAGAACGAGCACATAAGG |
| *atpF* R | GATTACCTGGGCTTCCGCTTTC |
| *cyoA* F | ATGGACTGGAAATGGTTCTTCATCTAC |
| *cyoA* R | TCATCACGGAGTTGGAGGTCAC |
| *cyoB* F | TCACCACAATGATTATCGCCATCC |
| *cyoB* R | GGTCCACAGCATCGCAGAATG |
| *mglB* F | TTCAACAAAGAACCGTCTCGTAAGG |
| *mglB* R | GCCCAGTGTTTAGCAATCAAATCG |
| *mglA* F | AGTTATGTGATGAAGTTACCGTATTGC |
| *mglA* R | GATCTTGTCCATCGTCAGTCCTG |
| *artI* F | TTGTGGGTCAGCAAGGCAAATAC |
| *artI* R | TGGTGTGTCGTCCCGTTCTG |
| *arnA* F | TATGGTTCGGTGGCTCGTCTG |
| *arnA* R | GGCGACAGTTGGGCAATGC |
| *arnC* F | CTTACCACAACGCCGCTACG |
| *arnC* R | CACCAGCAGCACCGCAATG |
| *arnT* F | AGATTTGTCAGCGGTGATGAGTTC |
| *arnT* R | GCGGAATGGCGAGACTGTTG |
| *arnD* F | CCATGCCGTTCCGTCCATTG |
| *arnD* R | TTTCACATCCCGACCAATCACTTC |
| *phoP* F | CGGTGCTGATGATTATGTGACTAAAC |
| *phoP* R | GGGAGCGAAATGACCTGTGAAG |
| *ompT* F | TACATTGGCTTGACTGGAAGTTATCG |
| *ompT* R | CGGGTCATAGTGTTCATCGTTATCAG |
| *danE* F | CGCGTTGATGCCAGAATAC |
| *danE* R | GCCGTTGATCTTGAACGAAC |
